# Soluble guanylate cyclase modulators—a novel therapeutic approach for diabetic kidney disease

**DOI:** 10.1080/0886022X.2025.2595385

**Published:** 2026-01-04

**Authors:** Yuqing Xu, Xuan Wu, Mingpai Ge, Kunhan Liang, Jixin Xing, Peng Zhang, Huilin Li, Zhiyong Guo, Xiaobin Mei

**Affiliations:** a Department of Nephrology, Changhai Hospital, Naval Medical University, Shanghai, China; b Department of Gastrointestinal Surgery, Changhai Hospital, Naval Medical University, Shanghai, China; c Department of Nephrology, Gongli Hospital of Shanghai Pudong New Area, Shanghai, China

**Keywords:** sGC modulators, diabetic kidney disease, proteinuria, efficacy, safety

## Abstract

Diabetic kidney disease (DKD) is a common complication, affecting approximately 40% of patients with diabetes and imposing substantial health and economic burdens worldwide. As DKD prevalence rises, existing treatments remain limited in efficacy, necessitating innovative therapeutic strategies. Soluble guanylate cyclase (sGC) modulators, including activators and stimulators, have emerged as promising treatments by enhancing cyclic guanosine monophosphate production, thereby exerting vasodilatory, anti-inflammatory, and anti-fibrotic effects. Preclinical studies have demonstrated their efficacy in improving renal function and reducing proteinuria in DKD models. Notably, clinical trials involving sGC activators, such as avenicguat, suggest potential benefits in managing DKD, particularly in combination with renin-angiotensin-aldosterone system inhibitors. While initial findings indicate renal protection and good tolerability, further large-scale studies are needed to confirm long-term efficacy and safety. The development of sGC modulators offers hope for improving DKD outcomes and reducing its global healthcare burden.

## Graphical abstract

**Figure:**
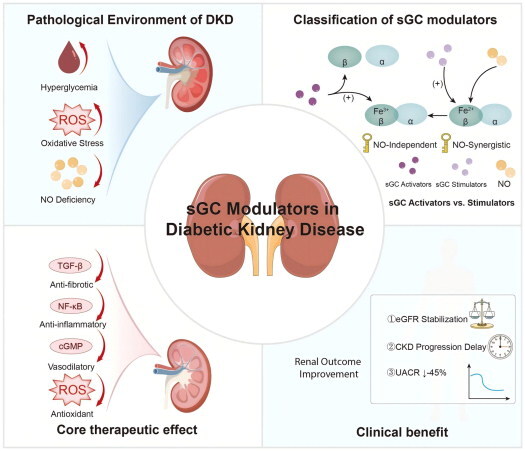

## 1. Introduction

According to the International Diabetes Federation, in 2021, the global number of patients with diabetes reached approximately 537 million. With an estimated prevalence rate of around 40%, approximately 215 million patients with diabetes worldwide may develop diabetic kidney disease (DKD). DKD significantly impacts health and quality of life and imposes substantial economic and resource burdens on global healthcare systems [1,2]. The pathogenesis of DKD is complex, with hyperglycemia inducing metabolic disturbances, inflammatory responses, hemodynamic changes, and fibrosis, all contributing to progressive kidney damage [3]. Although treatments such as renin–angiotensin–aldosterone system (RAAS) inhibitors, novel antidiabetic drugs like sodium–glucose cotransporter 2 inhibitors (SGLT2i) and glucagon-like peptide-1 receptor agonists (GLP-1RA), and finerenone have shown efficacy in controlling blood glucose and blood pressure, their limitations in slowing DKD progression remain evident. This underscores the urgent need for more effective therapeutic strategies [4].

Dysregulation of the nitric oxide (NO)–soluble guanylate cyclase (sGC)–cyclic guanosine monophosphate (cGMP) signaling pathway is a key feature of DKD pathophysiology. Restoring NO bioavailability or directly targeting sGC to increase cGMP levels may help mitigate DKD-associated pathological changes [5,6]. Traditional NO donor drugs (e.g. nitrates) have limited application in DKD due to tolerance and side effects. In contrast, novel sGC modulators (including sGC stimulators and activators) can directly activate sGC in an NO-independent manner, increasing cGMP levels and exerting anti-inflammatory, antifibrotic, and renal hemodynamic-improving effects [7,8]. This emerging therapeutic strategy offers hope for patients with DKD and may reduce its global burden. The following sections discuss the pathophysiological role of the NO–sGC–cGMP signaling pathway in DKD and the potential therapeutic value of sGC modulators.

## 2. Pathophysiology of the NO–sGC–cGMP pathway in DKD

sGC is a heterodimeric enzyme composed of α and β subunits that bind to NO molecules, activating their catalytic activity. sGC is present in the cytoplasm and acts as an intracellular NO receptor, which is synthesized from l-arginine by NO synthase (NOS). When NO binds to the heme domain of sGC, it induces a conformational change that stimulates the conversion of guanine triphosphate to cGMP, activating various downstream effector molecules, including cGMP-dependent protein kinase G, phosphodiesterases, and cyclic nucleotide-gated channels. The NO–sGC–cGMP pathway is involved in various physiological processes, including platelet aggregation and smooth muscle relaxation [9], regulating cardiovascular function while exerting anti-inflammatory, antithrombotic, and smooth muscle relaxation effects [10].

The physiological mechanisms by which sGC exerts its effects are diverse and play a critical role in maintaining normal renal function. Under physiological conditions, sGC is essential for preserving the glomerular filtration rate (GFR). First, NO-induced activation of sGC stimulates cGMP production, leading to vascular smooth muscle relaxation. This effect is particularly pronounced in the afferent and efferent arterioles of the glomerulus, enhancing renal blood flow [11]. Secondly,in the renal tubules—especially the proximal tubules and collecting ducts—sGC regulates sodium and water transport *via* the NO–cGMP signaling pathway, either through protein kinase G (PKG) activation or modulation of ion channels, such as sodium and potassium channels. These actions maintain fluid and electrolyte balance, promote sodium excretion, and reduce blood volume and pressure, which are essential for systemic blood pressure regulation [12]. Additionally, in the juxtaglomerular apparatus (JGA), sGC suppresses renin release *via* the NO–cGMP pathway, reducing RAAS activity. This renin secretion regulation is particularly significant in the kidney, as the JGA is the primary site of renin production. By directly influencing renin levels, sGC profoundly influences renal hemodynamics and systemic blood pressure [13].

Moreover, NO exerts additional effects by directly modifying proteins through mechanisms such as S-nitrosylation and indirectly regulates protein activity, influencing cellular signaling pathways. NO modulates immune cell phenotypes and functions, suppressing inflammatory responses. Furthermore, NO inhibits angiotensin II (Ang II) signaling and sympathetic nervous system activity, reducing vasoconstriction and inflammation [14]. Furthermore, NO protects cells from oxidative damage by inhibiting nicotinamide adenine dinucleotide phosphate hydrogen (NADPH) oxidase activity, enhancing antioxidant enzyme activity, and regulating mitochondrial energy metabolism, ultimately decreasing reactive oxygen species (ROS) production [15].

In hyperglycemic conditions, NO production is often impaired, decreasing sGC activity and reducing cGMP levels. Impairment of the NO–sGC–cGMP pathway results in glomerular capillary hypertension and reduced renal blood flow, contributing to renal dysfunction [16,17]. Furthermore, in DKD, there is generally a high level of oxidative stress, and elevated ROS levels can react with NO to form peroxynitrite, thereby reducing NO bioavailability. Endothelial dysfunction in a hyperglycemic environment further diminishes NO release and increases pro-inflammatory factors, leading to proteinuria and a hyperfiltration state in the glomeruli, thereby exacerbating renal injury [3,18,19].

## 3. sGC stimulators and activators

Based on their molecular mechanisms, sGC modulators are classified into two types: heme-dependent sGC stimulators and heme-independent sGC activators. The difference is that sGC stimulators require heme and can either directly stimulate sGC independent of NO or synergize with endogenous NO. Subsequently, the sGC stimulator stimulates sGC together with the reduced heme-iron complex [20]. These drugs play important roles in vasodilation and immune response. One notable sGC stimulator, riociguat, is primarily used to treat chronic thromboembolic pulmonary hypertension and pulmonary arterial hypertension. It effectively reduces pulmonary arterial pressure, increases cardiac output, and improves exercise capacity [21,22].

**Figure 1.**
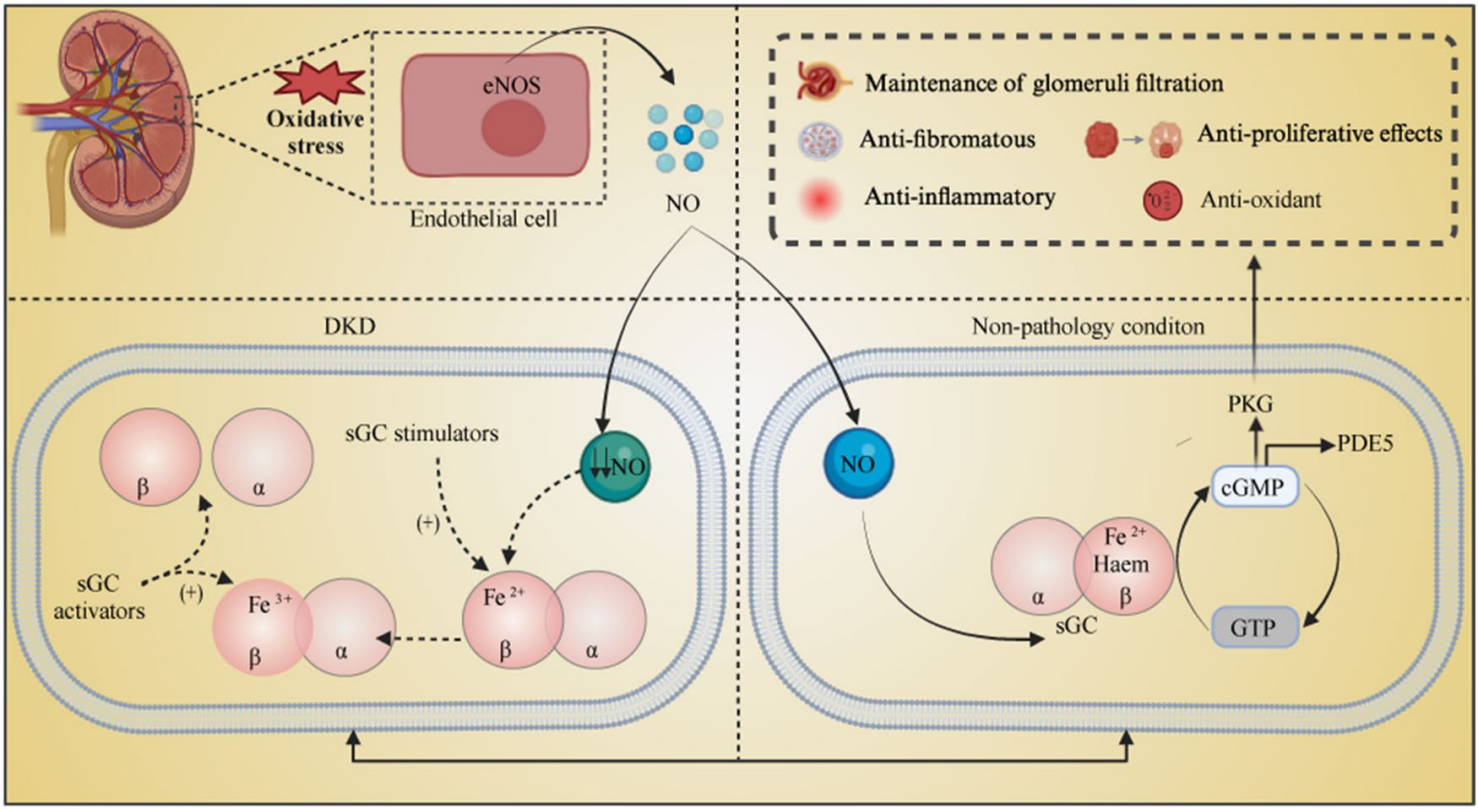
Pathophysiology of the NO–sGC–cGMP Pathway in DKD. In a physiological state, NO released by renal endothelial and epithelial cells stimulates sGC in smooth muscle cells, converting GTP into cGMP. However, in DKD, due to the high-glucose environment and oxidative stress imbalance, the activity of nitric oxide synthase (NOS) is affected, leading to a reduction in NO, which may be accompanied by sGC oxidation and/or degradation. There are two categories of sGC modulators: sGC stimulators bind to the heme group in its reduced state (Fe^2+^), increasing cGMP levels, while sGC activators can act on the oxidized state (Fe^3+^) and even in the absence of the heme group. Both types of sGC modulators can prevent sGC reduction, stimulating the activation of the NO/sGC/cGMP pathway. cGMP exerts its physiological functions, such as antioxidative stress, anti-inflammatory effects, endothelial repair, and restoration of normal renal filtration function, by activating downstream effector proteins like PKG.

In contrast, sGC activators do not require heme cofactors and primarily enhance the NO–sGC binding state, increasing intracellular cGMP levels even in the absence of NO. Oxidation of sGC further enhances the efficacy of activators. Therefore, when NO production or bioavailability declines due to oxidative stress or when phosphodiesterase type 5 (PDE5) inhibitors become ineffective, sGC activators may serve as an alternative therapeutic option [23]. The structure and activity of sGC modulators have also improved. First-generation sGC activators, such as cinaciguat and ataciguat, were discontinued due to poor pharmacokinetic properties and formulation limitations. Compounds like runcaciguat and avenciguat have been developed to address these limitations [24]. For instance, avenciguat (synonym: BI568809), as an sGC activator, demonstrates significant advantages in treating metabolic diseases such as DKD, obesity-related disorders, and cardiovascular complications, particularly under oxidative stress, where its efficacy may surpass that of sGC stimulators [25]. Although their mechanisms differ, both sGC modulators ultimately aim to induce vasodilation, lower blood pressure, and exert anti-inflammatory and antioxidant effects by increasing cGMP levels, offering novel insights into DKD treatment (Figure 1).

## 4. Preclinical studies of sGC modulators

Renal dysfunction is a common comorbidity in patients with chronic heart failure due to the interdependence between the heart and kidneys. In cardiovascular diseases (CVDs) research, including heart failure and hypertension, studies have revealed that sGC stimulators and activators also have protective effects on the kidneys. Consequently, interest has shifted toward exploring the relationship between sGC and cardiorenal metabolic syndrome (Table 1).

**Table 1.** Preclinical studies of sGC modulators.

Drug name	Model	Findings	Citation
Cinaciguat	eNOS KO+STZ	Cinaciguat treatment significantly improved GFR, serum creatinine, mesangial expansion, and renal fibrosis.	Harloff M, et al. [26]
Praliciguat	BMP rats	Improved proteinuria in diabetic nephropathy rats and validated tubular inflammation reduction at the cellular level.	Liu G, et al. [27]
	Dahl model	Reduced the expression of TGFβ and the main component of type I collagen, Col1α1, in the kidneys of mice, alleviating proteinuria.	Shea CM, et al. [28]
Riociguat	UUO	Riociguat reduced the expression of collagen-1, TGF-β, CTGF, α-SMA, and vimentin along with transcription factors, including Snail and Slug.	Sravani S, et al. [29]
Olinciguat	ZSF1 rats	Olinciguat has renal protective effects and is associated with lower circulating glucose, cholesterol, and triglycerides.	Zimmer DP, et al. [30]
Runcaciguat	ZSF1 rats	Significantly reduced proteinuria lowered kidney injury markers, improved renal structural damage, and increased survival rates, as well as kidney and heart injury markers.	Kraehling JR, et al. [31]
BI 703704	ZSF1 rats	BI703704 treatment led to a dose-dependent reduction in proteinuria, kidney weight, glomerulosclerosis, and interstitial fibrosis degree.	Boustany-Kari CM, et al. [32]
US 9365574B2	ZSF1 rats	Combined with enalapril, normalized blood pressure levels significantly reduced UPCR and UACR and improved GFR.	Hu L, et al. [33]
BAY 41-2272 BAY 60–2770	ApoE-/- mice	Indicates that sGC activators are more effective than sGC stimulators in treating diabetes-related vascular and renal complications.	Sharma A, et al. [34]

Although early research on sGC modulators focused on CVD treatment, the pathophysiological effects of endothelial dysfunction are quite extensive, affecting major organs, including the heart, kidneys, lungs, and cerebral vasculature [35,36]. Current research on sGC modulators for DKD remains limited, with some drugs still in preclinical experimental stages.

### 4.1. Cinaciguat

Cinaciguat, an sGC activator, was evaluated for its effects on DKD by Harloff et al. who treated streptozocin (STZ)-induced type 1 diabetic mice with cinaciguat. Cinaciguat improved the GFR in diabetic mice, reduced serum creatinine levels, and decreased mesangial expansion [37]. It inhibited thrombospondin 1 upregulation under diabetic conditions and altered the expression of kidney damage and fibrosis-related proteins, including collagen, fibronectin, and α-smooth muscle actin (α-SMA), as well as enzymes involved in extracellular matrix degradation such as matrix metalloproteinase-2 and tissue inhibitor of metalloproteinases-1 [26,38]. To enhance targeting, a study encapsulated cinaciguat in virus-mimicking nanoparticles that specifically target renal mesangial cells. This delivery method improved sGC stabilization, activation, and downstream signaling 4–5 times *in vitro*. Additionally, after administration, cinaciguat significantly inhibited the transforming growth factor beta (TGF-β) signaling pathway, reducing renal fibrosis [39]. These findings highlight cinaciguat’s potential to enhance kidney function and mitigate fibrosis.

### 4.2. Praliciguat

ZSF-1 rats are widely used as experimental models for metabolic syndromes, including diabetes, hypertension, and obesity [40]. The sGC stimulator praliciguat improved proteinuria in ZSF-1 rats and reduced inflammation and apoptosis in renal tubular epithelial cells [27,28,41]. Combined praliciguat–enalapril treatment showed greater efficacy than either agent alone [42]. Praliciguat has demonstrated renoprotective effects in hypertension-related kidney disease and renal fibrosis with minimal blood pressure impact [43]. Notably, praliciguat shows potential in improving kidney fibrosis in unilateral ureteral obstruction (UUO) mice by reducing the expression of pro-fibrotic factors such as TGF-β and collagen I, inhibiting inflammation and fibrosis, and restoring normal haemodynamic function [27]. Furthermore, combined praliciguat–empagliflozin therapy in high-fat diet-induced hypertensive rats proved to be more effective than the administration of either drug alone in terms of their effects on blood pressure, glucose and lipid metabolism, and kidney function, possibly due to their enhanced anti-inflammatory effects [44].

### 4.3. Riociguat

A study in Germany induced a DKD animal model using STZ in endothelial NOS (eNOS) knockout mice, where a combination of the sGC stimulator riociguat and angiotensin receptor blockers (ARBs) telmisartan reduced urinary protein excretion. Although telmisartan alone lowers blood pressure, its combination with riociguat significantly reduces urinary albumin excretion, approaching normal levels. Additionally, combination therapy reduces malondialdehyde immunoreactivity, an oxidative stress marker, suggesting that sGC stimulators may be beneficial for patients with poor response to standard ARB therapy [29,45]. In diabetic eNOS knockout mice, riociguat also reduced pro-inflammatory cytokine secretion and fibrotic factor expression, supporting its role in improving kidney injury and fibrosis [46].

### 4.4. Olinciguat

In a TNF-α-induced inflammation model, sGC stimulator olinciguat reduced soluble adhesion molecule levels produced by activated endothelial cells and leukocytes, suggesting its potential in preventing leukocyte-mediated vascular endothelial and tissue damage. Additionally, it has demonstrated cardiac and renal protective effects in animal studies and lowered blood pressure, glucose, cholesterol, and triglyceride levels [30,47].

### 4.5. Runcaciguat

In a study on ZSF1 rats, sGC activator runcaciguat significantly reduced proteinuria, HbA1c, triglyceride, and cholesterol levels, demonstrating renoprotective effects and alleviating inflammation and fibrosis. However, its impact on blood pressure was not significant, suggesting its therapeutic effect may be independent of blood pressure reduction [31,48].

### 4.6. Unnamed sGC modulators

In 2016, Boustany-Kari et al. applied the sGC activator BI 703704 to ZSF-1 rats to restore cGMP levels. After 15 weeks of treatment, a reduction in proteinuria, along with decreased incidence of glomerulosclerosis and interstitial lesions, was observed [32]. Research on another sGC stimulator, US 9365574B2, combined with enalapril, significantly lowered blood pressure, reduced the urinary protein-to-creatinine ratio (UPCR) and urinary albumin-to-creatinine ratio (UACR), and improved GFR [33]. A recent study demonstrated that the sGC activator BAY 60–2770 may inhibit the progression from acute kidney injury to chronic kidney disease (CKD), a previously understudied aspect, possibly by improving renal blood flow and tissue oxygenation [49]. Additionally, for the first time, a study compared two types of sGC modulators in diabetes-related vascular complications, highlighting the therapeutic advantages of sGC activators under oxidative stress conditions [34]. This supports the hypothesis that sGC activators function under oxidative stress or heme deficiency, whereas stimulators require low NO levels to enhance sGC activity. Since endogenous NO synthesis is significantly reduced in patients with diabetes, activators may offer a greater advantage.

## 5. Clinical studies of sGC modulators

Extensive animal studies have validated the therapeutic potential of sGC stimulators and modulators in DKD treatment, thereby advancing clinical research. Several large-scale clinical studies have investigated the potential benefits of these agents for kidney disease treatment (Tables 2 and 3). This section focuses on sGC modulators with the most comprehensive results.

**Table 2.** Clinical studies of sGC modulators.

Drug	Study population	Treatment	Main findings	Citation
Avenciguat	CKD with eGFR ranging from 20 to 90 mL/min/1.73 m² UACR between 200 and 3500 mg/g (*n* = 500). Patients with DKD and without DKD.	Avenciguat at 1 mg, 2 mg, and 3 mg TID for 20 weeks	Avenciguat significantly reduced UACR in all dosage groups, especially in the 3 mg TID group, which showed the greatest effect. The safety profile of avenciguat was good, with adverse events incidence during treatment similar to that of the placebo group	Heerspink HJL, et al. [50]
Praliciguat	Type 2 diabetes with eGFR ranging from 30 to 75 mL/min/1.73 m². UACR between 200 and 5,000 mg/g (*n* = 156). (All participants were receiving RAAS inhibitor treatments.)	Praliciguat at 20 mg or 40 mg QD for 12 weeks.	The changes in UACR for the praliciguat and placebo groups were 228% and 215%, respectively, which did not reach statistical significance. Praliciguat showed reductions in average 24-hour systolic blood pressure, HAb1c, and serum cholesterol.	Hanrahan JP, et al. [51]
Vericiguat	HFrEF with baseline eGFR ranging from 15 to 90 mL/min/1.73 m² (*n* = 4956)	2.5 mg orally once daily	Vericiguat reduced mortality in patients with heart failure, particularly in those with an eGFR of 30‒60 mL/min/1.73 m². Vericiguat did not reduce eGFR and creatinine levels.	Voors AA, et al. [52]
	Patients undergoing elective cardiac surgery (*n* = 170)	Vericiguat 10 mg orally starting 2 days before heart surgery through the day of surgery	Researchers will compare markers of kidney injury to see if vericiguat improves vascular function and reduces injury markers.	ClinicalTrials.gov ID NCT05812755Phase: Phase 4 Primary Completion (Estimated) 2026-10
Riociguat	HFpEFand PH with left ventricular ejection fraction greater than 50% (*n* = 46). (A diverse population with common comorbidities such as atrial fibrillation, diabetes mellitus, and chronic obstructive pulmonary disease.)	Riociguat at doses of 0.5 mg, 1 mg, or 2 mg	Riociguat increased stroke volume and cardiac index significantly while decreasing systolic blood pressure and right ventricular end-diastolic area. Changes in renal function (eGFR and creatinine levels) were similar between the riociguat and placebo groups.	Bonderman D, et al. [53]
Runcaciguat	Patients with CKD and established atherosclerotic cardiovascular disease or heart failure, plus T2D and/or hypertension, were enrolled. All were receiving stable maximum tolerated RAAS inhibitors with or without SGLT2i.(*n* = 243)	Runcaciguat once daily, titrated weekly (30–120 mg if tolerated).	UACR decreased by −45.2% versus placebo with runcaciguat in patients with CKD without SGLT2i (*p* < 0.001) and by −48.1% versus placebo in patients with CKD taking SGLT2i .	Gansevoort RT, et al. [54]

### 5.1. Avenciguat

Clinical studies indicate that the sGC activator avenciguat significantly reduces urinary albumin excretion in patients with DKD and CKD unrelated to diabetes when combined with ACEIs [55]. Randomized controlled trials demonstrate that avenciguat’s efficacy in reducing UACR is dose-dependent. At week 20, the placebo-corrected geometric mean changes in 10-h urinary UACR were reductions of 19.4%, 15.5%, and 23.4% in the 1 mg, 2 mg, and 3 mg TID groups, respectively. A similar trend was observed in morning urinary UACR, with a 23.4% reduction in the 3 mg TID group. Additionally, the proportion of patients achieving *a* ≥ 20% reduction in UACR also demonstrated a dose-dependent pattern, with 23%, 40%, and 48% of patients in the 1 mg, 2 mg, and 3 mg TID groups, respectively, compared to 27% in the placebo group. The odds ratio (OR) for achieving *a* ≥ 20% UACR reduction in the 3 mg TID group compared to placebo was 3.1. Furthermore, the UACR reduction in the 3 mg TID group was fully evident by week 6 and sustained through week 20. After discontinuing treatment for 4 weeks, UACR values gradually returned to baseline levels, indicating that the treatment effect is reversible. In addition, subgroup analysis showed that avenciguat’s efficacy was consistent, regardless of SGLT2 inhibitors use. Among patients using SGLT2 inhibitors, the 3 mg TID group had a 23.3% UACR reduction; in non-users, the reduction was 21.4% [50].

Avenciguat demonstrated good tolerability, with adverse event rates similar to that of the placebo group. Although mild-to-moderate adverse reactions, such as hypotension, diarrhea, and upper gastrointestinal bleeding, were observed in the treatment group during several clinical trials, most patients tolerated these reactions well, resulting in a low discontinuation rate. Therefore, when combined with ACEIs, avenciguat showed good efficacy in reducing urinary albumin excretion and acceptable safety, with no clinically significant pharmacokinetic interactions observed in co-administration studies. Notably, the synergistic RAAS-sGC pathway modulation allowed sustained BP control without exacerbating hyperkalemia risk even in advanced CKD stages. Moreover, a study assessed avenciguat’s safety, tolerability, pharmacokinetics, and pharmacodynamics following single ascending doses or multiple ascending doses in healthy volunteers. The drug was well tolerated, though high doses (5.0 mg) increased orthostatic hypotension incidence. However, most cases were mild to moderate, and the incidence was within a manageable range. Additionally, multiple doses (e.g. TID) improved tolerability and reduced the incidence of orthostatic hypotension. Furthermore, avenciguat was rapidly absorbed, reaching steady-state levels within 3–5 days, and TID dosing increased overall drug exposure without significantly raising peak plasma concentrations [56–58].

### 5.2. Praliciguat

The sGC stimulator praliciguat enhances NO signaling. Animal studies have shown significant improvements in proteinuria and renal fibrosis. In a 14-day, randomized, double-blind, placebo-controlled trial, 26 participants with type 2 diabetes and hypertension received various praliciguat doses. Results indicated a positive trend in reducing fasting blood glucose, total cholesterol, and low-density lipoprotein cholesterol levels, with notable improvements in blood pressure control, particularly in patients with higher baseline blood pressure. Although praliciguat demonstrated good tolerability and positive metabolic effects in the short term, its impact on renal function indicators, including UACR, remains unclear. Only one serious adverse event (upper gastrointestinal bleeding) was observed; however, the overall adverse event incidence was low [51]. This suggests that praliciguat is relatively safe for clinical application, though careful monitoring remains necessary.

In a 12-week trial, 156 participants with type 2 diabetes and moderate-to-severe renal impairment were randomly assigned to receive 20 or 40 mg praliciguat. Although praliciguat did not significantly outperform placebo in reducing UACR (*p* = 0.17), it improved blood pressure, glycated hemoglobin, and cholesterol levels, with more pronounced effects in the 40 mg dose group. The main findings suggest that praliciguat does not significantly reduce UACR in the short term. However, its positive effects on blood pressure, glycaemic control, and lipid levels highlight its potential for DKD management. Although upper gastrointestinal bleeding occurred in the high-dose group, praliciguat was well tolerated [59].

These findings warrant further investigation in larger clinical trials to assess praliciguat’s long-term efficacy and safety. Additionally, studies suggest that praliciguat serves as an adjunctive treatment in DKD management [51]. ACEI/ARB and SGLT2 inhibitors significantly improve urinary albumin excretion and slow down renal function decline, making them important choices for the treatment of DKD. Although praliciguathas shown metabolic and blood pressure benefits, its renal protective effects need further validation. It is worth noting that in the phase II trial, when used in combination with ACEI, praliciguat showed a significant reduction in UACR while maintaining considerable safety, indicating that the synergistic regulation of the RAAS sGC pathway does not exacerbate the risk of hypotension [60]. Overall, pralicguat is expected to be used in DKD research, supporting further exploration of large-scale trials. Combining the results of emerging therapies such as SGLT2 inhibitors and mineralocorticoid receptor modulators, this may provide more effective treatment options for DKD patients. Future research should focus on the long-term efficacy, safety, and impact on renal function of praxiwete, in order to expand the avenues for treating DKD.

### 5.3. Runcaciguat

A multicenter, double-blind, randomized, placebo-controlled phase 2a clinical trial enrolled 243 patients with chronic kidney disease at 82 centers. The study evaluated the efficacy and safety of the soluble guanylate cyclase activator runcaciguat in CKD patients with atherosclerotic cardiovascular disease or heart failure, with or without type 2 diabetes (T2D), all receiving maximally tolerated doses of renin-angiotensin system inhibitors (with or without SGLT2 inhibitors). Participants were randomized in a 3:1 ratio to receive runcaciguat (titrated from 30 mg up to 120 mg) or placebo for 8 weeks. The main outcome was the change in urinary albumin-to-creatinine ratio. Results showed that runcaciguat significantly reduced UACR in diabetic CKD patients, both with and without SGLT2 inhibitors—by about 45%, while the placebo group showed no improvement. In non-diabetic CKD patients, runcaciguat also lowered UACR, but the difference with placebo was not statistically significant. The reduction in UACR appeared early and was sustained during treatment, rebounding after stopping the drug. Runcaciguat was generally well tolerated, with slightly more mild-to-moderate side effects compared to placebo (most often peripheral edema), and no increase in serious adverse events. There was a mild, reversible decrease in eGFR, possibly related to lowered intraglomerular pressure, but no increase in cardiovascular events or acute kidney injury [54]. In summary, runcaciguat significantly reduced proteinuria in CKD patients, especially those with T2D, with good tolerability, supporting its potential as a new kidney protective therapy, though larger and longer-term studies are needed to confirm these findings [54,61].

### 5.4. Vericiguat and riociguat

Vericiguat and riociguat, as sGC stimulators, have been evaluated in previous studies for their potential renal protective effects in patients with CVDs. Vericiguat has been approved since 2021 for heart failure treatment. In the vericiguat Global Study in Subjects with HFrEF (VICTORIA) trial, vericiguat’s impact on renal function in patients with worsening heart failure and reduced ejection fraction was similar to that of the placebo group, without demonstrating significant renal benefits [52,62]. Although evidence of renal protection is lacking, its cardiovascular benefits may indirectly slow eGFR decline [63].

Similarly, studies on riociguat in pulmonary arterial hypertension and sickle cell disease have explored its effects on the kidneys. While it may enhance renal blood flow by reducing systemic and glomerular filtration pressures, clinical findings on renal outcomes remain inconclusive [53]. In a study on patients with sickle cell disease, riociguat showed a negligible trend toward reducing proteinuria, with a slight eGFR decline in the riociguat group compared to the placebo group [64]. This suggests that riociguat may have potential adverse effects on kidney function, though further research is needed to clarify its renal effects.

## 6. Quality assessment for inclusion in the study

To systematically evaluate the reliability of existing evidence, we conducted a methodological quality assessment of the included clinical studies using the Newcastle Ottawa Scale (Table 4). This scale classifies the risk of research bias based on randomization methods, allocation concealment, blinding implementation, data integrity, and selective reporting. Ultimately, the research quality is classified as A (low bias), B (moderate bias), and C (high bias).

**Table 3.** Comparative pharmacological profiles of sGC modulators in diabetic kidney disease: mechanistic class, clinical efficacy, pharmacokinetics, and safety stratification.

Parameter	Avenciguat (Activator)	Praliciguat (Stimulator)	Runcaciguat (Activator)	Riociguat (Stimulator)
Mechanistic class	Heme-independent activator	Heme-depend ent stimulator	Heme-independent activator	Heme-dependent stimulator
Key targets	Oxidized/heme-free sGC	Reduced heme-bound sGC	Oxidized sGC	Reduced heme-bound sGC
Clinical efficacy				
UACR reduction*	23.4% (3 mg TID) [51]	NSD (40 mg QD) [56]	45.2% (120 mg QD) [44]	−6.5% (2.5 mg BID) [62]
SBP reduction	3.2 mmHg [53]	8.1 mmHg [55]	−2.5 mmHg [44]	−4.8 mmHg [61]
Pharmacokinetics				
Tmax (h)	2.0-3.5 [53]	1.5-2.0 [55]	4.0-6.0 [24]	1.0-1.5 [21]
Protein binding	92% [53]	85% [55]	89% [24]	95% [21]
Safety profile				
Hypotension risk	++ (Dose-dependent) [51]	+ [56]	+ [44]	+++ (PAH trials) [21]
GI adverse events	18% (3 mg) [51]	23% (40 mg) [56]	12% [44]	9% [61]
Therapeutic niches	CKD with high oxidative stress	Early DKD with preserved NO	Advanced DKD with heme depletion	PAH-associated renal dysfunction

**Table 4.** Newcastle Ottawa scale (NOS).

Includedin the study	Stochastic method	Allocation concealment	Party	Blind method The evaluator	Result Data integrity	Selective bias	Other biases	Quality Grade
Heerspink HJL [50]	Low risk	Low risk	Low risk	Low risk	Low risk	Low risk	Low risk	A level
Hanrahan JP [51]	Low risk	Low risk	Low risk	Low risk	Low risk	Low risk	Low risk	A level
Voors AA [52]	NK	NK	NK	NK	Low risk	NK	Low risk	B level
Bonderman D [53]	Low risk	Low risk	Low risk	Low risk	Low risk	Low risk	Low risk	A level

**Table 5.** Comparative efficacy and target populations.

Parameter	Stimulator (Riociguat)[64]	Activator (Avenciguat)[51]
Action site	Heme-bound state (Fe²⁺)	Oxidized/heme-free state
Efficacy under oxidative stress	↓ (NO-dependent)	↑ (NO-independent)
Target population	Early DKD (preserved NO synthesis)	Advanced/high-oxidative-stress DKD

As shown in Table 4, the randomized controlled trials conducted by Heerspink et al. [50] and Hanrahan et al. [51] demonstrated low risk in terms of random sequence generation, allocation concealment, double-blind design, and result data integrity, receiving an A-level rating. These two studies used a central randomization system, implemented strict blinding on subjects, researchers, and outcome assessors, and had a follow-up completion rate of over 95%. The UACR and eGFR results were highly reliable. However, Voors et al. [52] rated the study on Vilsigil as Grade B due to the potential risk of selective reporting of blood pressure outcomes, as the randomization details and blinding procedures were not clearly described. Although Bonderman et al. ‘s [53] study on levocetirizine was a small sample exploratory trial, it fully reported hemodynamic and renal safety indicators, which still constitutes low bias evidence.

It is worth noting that none of the studies explicitly stated the implementation of blinding for laboratory testing personnel, which may introduce detection bias in the measurement of biomarkers such as UACR. In addition, over 60% of the studies did not disclose their statistical analysis plans, which poses a risk of overinterpretation in *post hoc* subgroup analysis. These methodological limitations suggest that the current evidence for the renal protective effects of sGC modulators is of moderate strength (GRADE rating: B), and further pre registered, blinded phase III clinical trials are needed for validation.

We summarizes the pharmacokinetic characteristics and drug interaction risks of two sGC modulators. Riociguat (stimulant) is mainly metabolized by CYP3A4 enzyme, and caution should be exercised about the potential risk of drug accumulation when combined with potent CYP3A4 inhibitors (such as ketoconazole); The main metabolic pathway of avenciguat (activator) relies on UGT1A9 enzyme, and special attention should be paid to its interaction with UGT1A9 inhibitor probenecid, which may alter drug clearance rate. This table emphasizes the need to evaluate drug compatibility contraindications based on metabolic pathways during clinical medication, especially for patients with liver dysfunction or receiving multi drug therapy. Blood drug concentrations should be monitored or dosages adjusted to avoid toxic reactions. Future research needs to focus on the long-term safety of sGC modulators, especially the effects of different metabolic pathways (such as CYP3A4 and UGT1A9) on drug clearance (Tables 5 and 6). In clinical practice, the dosage should be adjusted according to the patient’s liver function and concomitant medication (such as ketoconazole and probenecid) to avoid toxic reactions.

**Table 6.** Pharmacokinetic interactions and clinical risk mitigation strategies.

Drug	Main Metabolizing Enzyme	High-risk drugs for Interaction
Riociguat	CYP3A4	Potent Inhibitors (Ketoconazole)
Avenciguat	UGT1A9	Probenecid
Drug	Main Metabolizing Enzyme	High-risk Drugs for Interaction

Concomitant use with NO donors or PDE5 inhibitors may precipitate severe hypotension. Avoid in hemodynamically unstable patients. Monitor BP closely if combination is unavoidable.

While the short-term safety profile of avenciguat appears favorable in phase II trials, the long-term consequences of chronic sGC modulation warrant cautious consideration. Prolonged systemic vasodilation through cGMP signaling may lead to cumulative hypotensive effects, particularly in elderly patients with autonomic dysfunction [65]. Furthermore, sustained activation of the NO-sGC-cGMP pathway could theoretically disrupt endogenous redox homeostasis, as observed in animal models where compensatory upregulation of phosphodiesterase-5 (PDE5) activity paradoxically diminished therapeutic efficacy after 6 months of continuous sGC activation [66]. Of particular concern is the potential for cross-talk between cGMP and cAMP pathways in renal tubular cells, which may induce electrolyte disturbances (e.g. hypokalemia) through enhanced Na+/K+-ATPase activity when co-administered with diuretics [43]. These mechanisms highlight the need for extended follow-up studies to evaluate renal and cardiovascular outcomes beyond 2 years of treatment.

Co-administration of sGC modulators with NO donors (e.g. nitroglycerin) or PDE5 inhibitors (e.g. sildenafil) may induce synergistic hypotension due to amplified cGMP accumulation [21,67]. This risk is heightened in elderly patients or those with advanced CKD (eGFR <30 mL/min/1.73 m^2^), where impaired drug clearance and autonomic dysfunction exacerbate hemodynamic instability. Dose titration and real-time blood pressure monitoring are mandatory in such scenarios, especially during therapy initiation. For instance, riociguat requires a 50% dose reduction when combined with strong CYP3A4 inhibitors (Table 6), while avenciguat’s UGT1A9-mediated metabolism necessitates caution with probenecid co-administration.

## 7. Discussion

The role of sGC modulators in DKD treatment has gained increasing research attention. Current findings suggest that the primary mechanism of sGC modulators involves enhancing the NO–sGC–cGMP signaling pathway, thereby improving vascular function and exerting anti-inflammatory, antifibrotic, and antioxidant effects [11]. These actions show potential in alleviating DKD pathology, including reducing proteinuria, improving GFR, and inhibiting renal fibrosis. However, these effects primarily target downstream pathological mechanisms rather than directly addressing upstream causes such as hyperglycemia and insulin resistance (Table 1). Consequently, sGC modulators are currently viewed as more inclined to “treat the symptoms,” focusing on improving renal function and slowing disease progression rather than reversing the fundamental causes of the condition.

Nonetheless, this does not diminish the significance of sGC modulators. DKD pathogenesis is highly complex, and single-target therapies often fail to control disease progression sufficiently. The multi-target effects of sGC modulators, particularly their ability to combat oxidative stress and fibrosis, make them valuable as adjunct therapies. In the future, sGC modulators may play a more prominent role in combination treatment strategies, complementing existing therapies such as RAAS inhibitors and SGLT2 inhibitors to achieve more comprehensive therapeutic outcomes.

While the canonical NO-sGC-cGMP pathway underpins the primary mechanism of sGC modulators, emerging evidence suggests potential cGMP-independent effects that may contribute to their renal benefits. For instance, sGC conformational changes induced by activators like avenciguat could directly modulate protein-protein interactions (e.g. with HSP90) or influence redox-sensitive signaling cascades independent of cGMP production [43,66]. In advanced DKD stages characterized by severe oxidative stress and heme oxidation, cGMP-independent mechanisms—such as suppression of NF-κB-mediated inflammation or direct inhibition of TGF-β/Smad3 fibrotic signaling—may become clinically relevant [25,34]. Future studies should delineate the relative contributions of cGMP-dependent versus -independent pathways across DKD progression, particularly in cohorts resistant to RAAS inhibition.

The different results observed using praliciguat showed significant improvement in proteinuria in preclinical models, but no significant reduction in UACR was demonstrated in clinical trials, highlighting the key translational challenge in DKD treatment. Several factors may underlie this discrepancy: First, predominant use of type 1 diabetes models (STZ-induced mice) in preclinical studies inadequately replicates the complex metabolic milieu of human type 2 DKD, particularly regarding insulin resistance and dyslipidemia interactions. Second, the 12-week clinical trial duration may be insufficient to detect structural renal changes, contrasting with animal studies employing 15–20 week interventions [27,28]. Third, dose selection based on blood pressure effects rather than tissue cGMP target engagement might lead to subtherapeutic renal exposure [51]. Lastly, reliance on UACR as a surrogate endpoint may overlook praliciguat’s potential effects on tubular proteinuria components not captured by albumin-specific assays [60]. These translational gaps emphasize the need for humanized models incorporating diabetic comorbidities and extended clinical trials with kidney histology endpoints (Tables 3 and 5).

Between sGC stimulators and activators, the latter appears to have greater therapeutic potential. sGC activators directly activate sGC under oxidative conditions or in the absence of heme, remaining effective even when NO levels are depleted. This unique mechanism gives activators a significant advantage in patients with DKD who exhibit high oxidative stress levels. However, further research is needed to explore sGC modulator applications across DKD subtypes. For instance, stimulators may be more suitable for patients with preserved NO levels, enabling a more personalized treatment approach.

Although research on sGC modulators remains in its early stages, several promising directions warrant attention. One key area involves combining sGC modulators with existing therapies. sGC modulators enhance the NO–sGC–cGMP pathway, improving vascular function and exerting anti-inflammatory, antifibrotic, and antioxidant effects in DKD, but primarily target downstream mechanisms (e.g. proteinuria, renal fibrosis) rather than upstream drivers like hyperglycemia, necessitating adjunct use with RAAS/SGLT2 inhibitors for synergistic benefits [11]. Clinical trials have demonstrated that avenciguat, when combined with ACE inhibitors, significantly reduces proteinuria, while praliciguat, combined with empagliflozin, exhibits enhanced anti-inflammatory and renal protective effects in preclinical studies. Furthermore, advancements in targeted drug delivery systems have expanded possibilities for sGC modulator applications. Second-generation sGC activators, such as runcaciguat, aim to overcome the pharmacokinetic and formulation limitations of first-generation drugs, offering promising potential for future therapeutic applications [24]. In addition, clinical evidence indicates that cinaciguat exerts potent cardiovascular effects, reducing both preload and afterload [53]. However, research on its renal effects remains limited. As an sGC activator similar to avenciguat, the efficacy of cinaciguat in kidney diseases is highly anticipated [65,67].

The limitations of this study are mainly reflected in three aspects: preclinical models, clinical trial design, and risk of bias. Commonly used animal models (such as ZSF-1 rats) lack the typical pathological features of human DKD (such as glomerular nodular sclerosis), which may lead to an overestimation of the therapeutic effect [40]. The sample sizes of the phase II clinical trials were relatively small (praliciguat trial *n* = 156, avenciguat trial *n* = 500) and the follow-up durations were short (≤20 weeks), making it difficult to assess hard endpoints (such as end-stage renal disease) and long-term renal safety [50,51]. In addition, 83% of clinical studies are funded by pharmaceutical companies, which may lead to insufficient reporting of adverse events (such as the risk of hypotension with the first-generation sGC activator cinaciguat [64]. In the future, it is necessary to establish humanized models that integrate diabetes comorbidities (insulin resistance, lipid metabolism disorders), conduct phase III clinical trials lasting ≥1 year with renal histopathological endpoints, and directly compare the efficacy of sGC activators and stimulators at different stages of DKD. The focus should be on elucidating the mechanisms by which both agents regulate tubulointerstitial injury—the core prognostic factor.

## 8. Conclusion

sGC modulators enhance the NO-sGC-cGMP signaling pathway, improving vascular function and reducing inflammatory responses. Although avenciguat and runcaciguat demonstrate significant proteinuria reduction—a promising biomarker for early DKD—this effect has not been validated as a definitive surrogate for hard renal endpoints such as end-stage renal disease or 40% eGFR decline. Proteinuria reduction alone cannot justify therapeutic adoption without robust evidence from phase III trials with longitudinal follow-up (>3 years) confirming preservation of kidney function. Future studies must prioritize end-stage renal disease, cardiovascular mortality, and safety outcomes in advanced CKD populations to establish clinical utility.

sGC modulators enhance the NO–sGC–cGMP signaling pathway, improving vascular function and reducing inflammatory responses. They may also positively affect pathological processes such as renal fibrosis and oxidative stress. Although research remains in its early stages, the diversity of mechanisms and promising preclinical results offer hope for future clinical applications. Notably, the significant proteinuria reduction observed with avenciguat in patients with DKD underscores the potential of this drug class in improving renal function and slowing disease progression. With ongoing research and accumulating clinical data, sGC modulators are expected to emerge as a novel therapeutic breakthrough in DKD, providing patients with more effective treatment options. However, the translation of these benefits into long-term renal preservation requires rigorous validation through decade-spanning outcome studies, particularly regarding hemodynamic adaptation and off-target signaling cascades.

## Data Availability

Data sharing is not applicable to this article as no new data were created or analyzed in this study. As a review article, all discussed information and conclusions are based on previously published studies, which are cited in the reference list
